# Predictors of first-line antiretroviral therapy discontinuation due to drug-related adverse events in HIV-infected patients: a retrospective cohort study

**DOI:** 10.1186/1471-2334-12-296

**Published:** 2012-11-12

**Authors:** Mattia CF Prosperi, Massimiliano Fabbiani, Iuri Fanti, Mauro Zaccarelli, Manuela Colafigli, Annalisa Mondi, Alessandro D’Avino, Alberto Borghetti, Roberto Cauda, Simona Di Giambenedetto

**Affiliations:** 1 Clinical Infectious Diseases, Catholic University of Sacred Heart, Rome, Italy; 2 Emerging Pathogens Institute & Department of Pathology, Immunology and Laboratory Medicine, College of Medicine, University of Florida, Gainesville, Florida, USA; 3 Viral Immunodeficiency Unit, National Institute for Infectious Diseases “Lazzaro Spallanzani”, Rome, Italy; 4 Clinical Department, National Institute for Infectious Diseases “Lazzaro Spallanzani”, Via Portuense 292, 00149, Rome, Italy

**Keywords:** HIV, HAART, Toxicity, Side effects, Therapy-naïve

## Abstract

**Background:**

Drug-related toxicity has been one of the main causes of antiretroviral treatment discontinuation. However, its determinants are not fully understood. Aim of this study was to investigate predictors of first-line antiretroviral therapy discontinuation due to adverse events and their evolution in recent years.

**Methods:**

Patients starting first-line antiretroviral therapy were retrospectively selected. Primary end-point was the time to discontinuation of therapy due to adverse events, estimating incidence, fitting Kaplan-Meier and multivariable Cox regression models upon clinical/demographic/chemical baseline patients’ markers.

**Results:**

1,096 patients were included: 302 discontinuations for adverse events were observed over 1,861 person years of follow-up between 1988 and 2010, corresponding to an incidence (95% CI) of 0.16 (0.14-0.18). By Kaplan-Meier estimation, the probabilities (95% CI) of being free from an adverse event at 90 days, 180 days, one year, two years, and five years were 0.88 (0.86-0.90), 0.85 (0.83-0.87), 0.79 (0.76-0.81), 0.70 (0.67-0.74), 0.55 (0.50-0.61), respectively. The most represented adverse events were gastrointestinal symptoms (28.5%), hematological (13.2%) or metabolic (lipid and glucose metabolism, lipodystrophy) (11.3%) toxicities and hypersensitivity reactions (9.3%). Factors associated with an increased hazard of adverse events were: older age, CDC stage C, female gender, homo/bisexual risk group (vs. heterosexual), HBsAg-positivity. Among drugs, zidovudine, stavudine, zalcitabine, didanosine, full-dose ritonavir, indinavir but also efavirenz (actually recommended for first-line regimens) were associated to an increased hazard of toxicity. Moreover, patients infected by HIV genotype F1 showed a trend for a higher risk of adverse events.

**Conclusions:**

After starting antiretroviral therapy, the probability of remaining free from adverse events seems to decrease over time. Among drugs associated with increased toxicity, only one is currently recommended for first-line regimens but with improved drug formulation. Older age, CDC stage, MSM risk factor and gender are also associated with an increased hazard of toxicity and should be considered when designing a first-line regimen.

## Background

Combination antiretroviral therapy (cART) has markedly changed the prognosis of HIV-infected patients, reducing AIDS-related morbidity and mortality [1]. Rates of virological failure during first line regimens are decreasing both in clinical trials and in studies performed during routine clinical practice [2,3]. However, drug-related adverse events and toxicities are increasingly recognized [4-7] and represent one of the most common reasons for treatment discontinuation or switch [2,3,8-11]. In recent years, the introduction of newer antiretroviral agents with improved efficacy and tolerability profiles has allowed for a decline of treatment-limiting toxic effects; however, drug-related adverse events still represent an issue of concern.

Treatment-limiting cART toxicity has been associated with several factors such as demographical characteristics, drug-drug interactions, co-morbidities and recently genetic factors [12]. However, determinants of toxicity are not fully understood. In particular, the role of newer and apparently better tolerated antiretroviral drugs needs to be fully investigated. Moreover, the influence of baseline chemistry remains to be elucidated.

The aim of this study is to investigate predictors of first-line antiretroviral therapy discontinuation due to adverse events and their evolution in more recent years, characterized by an increased use of regimens with better tolerability profiles. In particular, the analysis is focused on the evaluation of baseline demographic and clinical characteristics, prescribed drugs and chemical parameters which could represent objective tools to tailor regimens on patients’ characteristics.

## Methods

HIV-infected patients followed up at the Infectious Diseases Clinic of the Catholic University of the Sacred Heart (CUSH) in Rome, Italy, starting a first-line anti-HIV antiretroviral therapy, were screened retrospectively via the electronic CUSH data base, which includes data on more than 4,300 HIV-infected patients, with the first available anti-HIV therapy record dated 1988. All patients included in the data base had previously signed an informed consent to be included in observational studies. Access and data analyses of the CUSH data base are regulated by an institutional internal ethics committee and conform to Italian and European privacy legislations. The latest available updated version (up to January 2011) of the CUSH data base was used.

The baseline time was the start date of the anti-HIV antiretroviral therapy. The end-point of interest was the discontinuation date of the first-line anti-HIV antiretroviral therapy. Discontinuation was defined as stopping any antiretroviral drug for at least 2 weeks or switching (changing one or more drugs) to another regimen; the only exception was a substitution of lamivudine with emtricitabine or vice-versa, because it could simply reflect a shift to a fixed dose formulation of tenofovir-emtricitabine, abacavir-lamivudine or zidovudine-lamivudine. An adverse event was marked if the reason of discontinuation or switch (or eventually a concomitant death) was an event corresponding to toxicity or allergy; otherwise data were censored at that time point or at the latest available time point if the patient did not stop that therapy, following a cause-specific approach. All discontinuation causes were ascertained by electronic and clinical reports (see Table 1 for categories besides the adverse event definition). In case of a decease event without a prior therapy discontinuation date, data were censored as well. First-line anti-HIV antiretroviral therapies with a known stop date, but unknown reason of stop or decease, were not included in the study population.

**Table 1:** Causes of anti-HIV first-line therapy discontinuation in the study population (n=1,096)

**Type**	**Tot**	**Strata**	**n**	**% Over strata**	**% Over total**
Adverse events (toxicity/allergy)	302 (1 death)	Gastrointestinal	86	28.5%	7.8%
		Hypersensitivity	28	9.3%	2.6%
		Central nervous system	18	6.0%	1.6%
		Hepatic	14	4.6%	1.3%
		Metabolic (glucose or lipid metabolism, lipodystrophy)	34	11.3%	3.1%
		Renal	13	4.3%	1.2%
		Hematologic	40	13.2%	3.6%
		Other	69	22.8%	6.3%
Other causes	618	Simplification	252	40.8%	23.0%
		Patient’s choice	93	15.0%	8.5%
		Failure	86	13.9%	7.8%
		Regimen intensification	59	9.5%	5.4%
		Genotype-guided switch	20	3.2%	1.8%
		Low adherence	19	3.1%	1.7%
		End of pregnancy	16	2.6%	1.5%
		Structured interruption	13	2.1%	1.2%
		Enrolment in a new prospective study	13	2.1%	1.2%
		Pregnancy	12	1.9%	1.1%
		Other	35	5.7%	3.2%
Non-discontinued	176 (6 deaths)	N/A	176	100%	16.1%

Covariates of interest, contemporary or the closest previous to the baseline date, were: calendar year, patient’s gender, age, nationality, risk group, first date of HIV-positive antibody test, CDC stage, viral subtype, anti-HIV antiretroviral therapy, HIV-RNA load, CD4^+^ T cell count, hepatitis C co-infection (HCVAb), hepatitis B co-infection (HBsAg), anti-HCV interferon/ribavirin treatment, anti-*Mycobacterium Tuberculosis* (TB) therapy, anti-*Pneumocystis jirovecii* pneumonia (PCP) therapy, other antibiotic treatments, other concomitant (prescription or over-the-counter) drugs exposure, total bilirubin, total cholesterol, hemoglobin, glucose, glutamate pyruvate transaminases (GPT), gamma-glutamyltransferase (gammaGT), high-density lipoprotein (HDL), low-density lipoprotein (LDL), triglycerides (TGL), and glomerular filtration rate (GFR) estimated by modification of diet in renal disease (MDRD) formula. Chemistry parameters were classified as normal, high or low according to established cut-offs [13,14] (see Table 2 for cut-off values). The covariate list was consistent with other European cohort studies [15,16]. Baseline chemistry variables were registered in the database only for patients starting antiretroviral therapy after 1998; when not available, they were encoded as unknown.

**Table 2:** Baseline chemical markers (with cut-offs) of the study population (n=1,096)

**Factor**	**Strata**	**n**	**%**
Bilirubin	Normal	624	56.9%
	High (>1.2 mg/dL)	31	2.8%
	Unknown	441	40.2%
Total Cholesterol	Normal	478	43.6%
	High (≥200 mg/dL)	107	9.8%
	Unknown	511	46.6%
Hemoglobin	Low (<12 g/dL)	158	14.4%
	Normal	593	54.1%
	Unknown	345	31.5%
GammaGT	Normal	453	41.3%
	High (>60 IU/L)	145	13.2%
	Unknown	498	45.4%
Glucose	Normal	674	61.5%
	High (>110 mg/dl)	34	3.1%
	Diabetes (>126 mg/dL)	22	2.0%
	Unknown	366	33.4%
GPT	Normal	561	51.2%
	High (>40 IU/L)	224	20.4%
	Unknown	311	28.4%
HDL	Low (<40 mg/dL)	217	19.8%
	Normal	121	11.0%
	Unknown	758	69.2%
LDL	Normal	306	27.9%
	High (≥130 mg/dL)	12	1.1%
	Unknown	778	71.0%
Tryglicerides	Normal	383	34.9%
	high (≥150 mg/dL)	178	16.2%
	Unknown	535	48.8%
MDRD	Low (<60 mg/dL/1.73m^2^)	20	1.8%
	Normal	694	63.3%
	Unknown	382	34.9%

The antiretroviral therapy was encoded in different ways: (i) as a combination therapy of 2 nucleoside reverse transcriptase inhibitors (NRTI) + 1 non-nucleoside reverse transcriptase inhibitor (NNRTI), 2 NRTI + 1 protease inhibitor (PI), 2 NRTI + 1 PI boosted with ritonavir (PI/r), and other combinations; (ii) as single compounds, i.e. emtricitabine, lamivudine, abacavir, zidovudine, stavudine, zaltacibine, didanosine, tenofovir, nevirapine, efavirenz, atazanavir, fosamprenavir, indinavir, lopinavir, nelfinavir, ritonavir full or boosting dose, and saquinavir; (iii) as a combination therapy of different brand names, including those combinations with a frequency of at least 10; (iv) as Truvada® + Sustiva®, Atripla®, Reyataz®, Kaletra®, any other NRTI+PI, any other NRTI+PI/r, any other NRTI+NNRTI, and other combinations. Other compounds including enfuvurtide, maraviroc, raltegravir, darunavir, and etravirine, were not included due to their low frequency.

Statistical methods included descriptive summaries, calculation of incidence ratios and confidence intervals, Kaplan-Meier estimation for the probability of surviving an adverse event, multivariable proportional-hazard hypothesis testing, and multivariable Cox model fitting in order to identify factors associated to an increased/reduced hazard of an adverse event happening. Effect of covariate interactions and nested model comparison via ANOVA were also carried out. All statistical analyses were carried out using R software (http://www.r-project.org).

## Results

**Figure 1:**
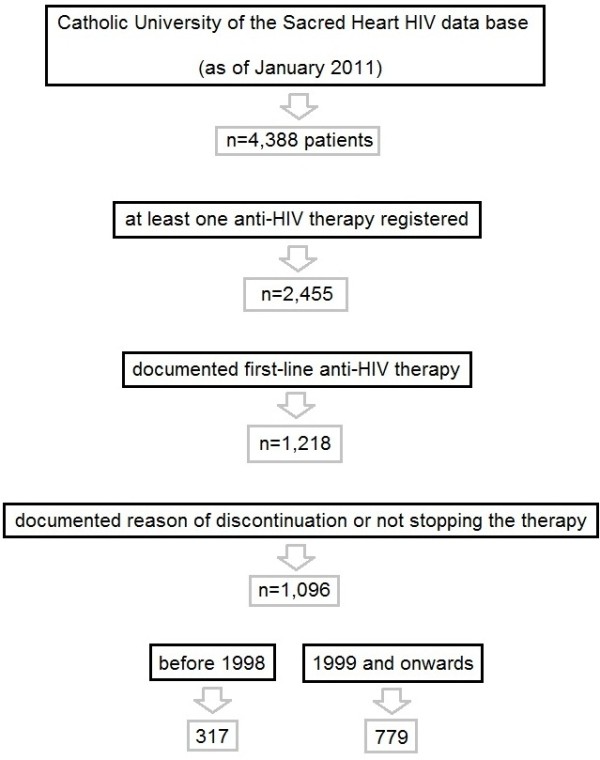
Flow chart of patients included in the study from the whole CUSH HIV data base.

The total number of HIV-positive patients recorded in the CUSH data base (up to January 2011) was 4,388. Those with at least one anti-HIV therapy registered were 2,455, and 1,218 for which it was the actual first-line. Of these, 1,096 had a documented reason of discontinuation or did not stop the therapy and met the inclusion criteria, starting their first-line anti-HIV therapy between year 1988 and 2010 (median 2003, interquartile range 1998–2006). Of these patients, 317 started the first-line anti-HIV therapy prior to 1999 (154 before 1997), whilst 779 afterwards (Figure 1). Tables 3 and 2 list patients’ demographic/clinical characteristics and chemical markers at the baseline date, concomitant to the first-line anti-HIV therapy start date. Also, Additional file 1 shows the distribution of patients under different first-line therapy regimens per calendar year, using both encoding (i) and (iv).

**Table 3:** Characteristics of the study population (n=1,096)

**Factor/strata**			**n**	**%**
Female gender			372	33.9%
Non Italian nationality			284	25.9%
Risk group	Heterosexual		421	38.4%
	Homo/bisexual		213	19.4%
	IDU		199	18.2%
	Other/unknown		263	24.0%
CDC stage	A		495	45.2%
	B		229	20.9%
	C		372	33.9%
HIV subtype	B		284	25.9%
	28_BF		84	7.7%
	17_BF		48	4.4%
	F1		27	2.5%
	Other		48	4.4%
	Unknown		557	50.8%
First-line anti-HIV therapy (encoding i)	2NRTI+1NNRTI		227	20.7%
	2NRTI+1PI		214	19.5%
	2NRTI+1PI/r		426	38.9%
	Other combination		229	20.9%
First-line anti-HIV therapy (encoding iv)	Truvada® + Sustiva®		47	4.3%
	Atripla®		14	1.2%
	Reyataz® ± ritonavir + backbone		22	2.0%
	Kaletra® + backbone		389	35.5%
	Any other NRTI+NNRTI		165	15.0%
	Any other NRTI+PI		221	20.2%
	Any other NRTI+PI/r		18	1.6%
	Other combinations		220	20.1%
Non-anti-HIV therapies (concomitant)	anti-TB		140	12.8%
	anti-PCP		425	38.8%
	anti-HCV		14	1.3%
	other antibiotics		194	17.7%
	other drugs		238	21.7%
HBsAg	negative		978	89.2%
	positive		56	5.1%
	unknown		62	5.7%
HCVAb	negative		771	70.3%
	positive		247	22.5%
	unknown		78	7.1%
Discontinuation/switch for adverse events			302	27.5%
Discontinuation/switch for other causes			618	56.4%
Non-discontinued first-line anti-HIV therapies			176	16.1%
**Years**	**1988-1993**	**1994-1998**	**1999-2004**	**2005-2010**
Discontinuation/switch for adverse events	17	92	108	85
Patients starting a first-line ART	88	229	349	430
PYFU	282.06	350.26	724.36	504.58
**Numerical markers**			**Median**	**IQR**
Calendar year			2003	1998-2006
Days on first-line anti-HIV therapy			379	131-855
Age (years)			36	31-43
Years from the first HIV positive test			0.5	0.1-3.4
HIV-RNA Log_10_ copies/mL			4.8	4.7-5.2
CD4^+^ T cells/mm^3^			194	122-233

**Figure 2:**
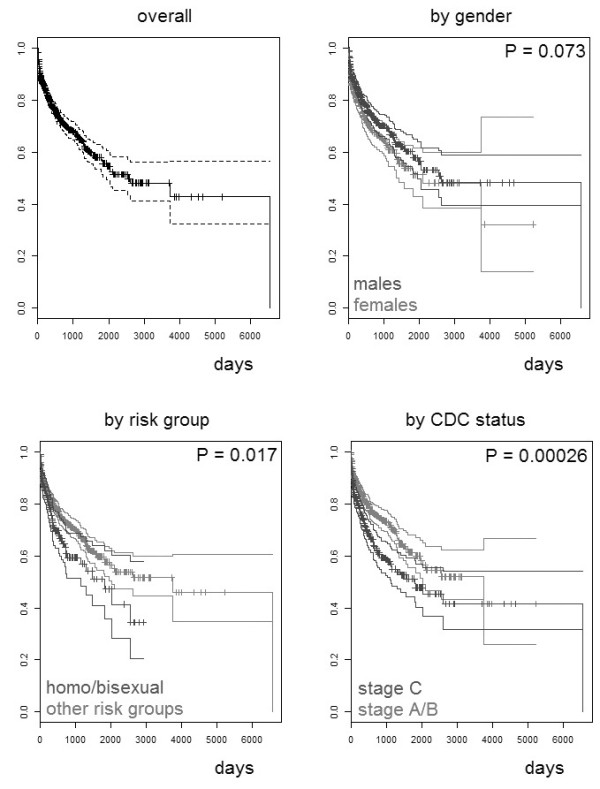
Kaplan-Meier estimations of the probability (with 95% confidence intervals) to be free of adverse events after initiating a first-line antiretroviral therapy.

The observed number of therapy discontinuations for adverse events (toxicity/allergy) was 302, over 1,861 person years of follow up, corresponding to an incidence (95% confidence intervals, CI) of 0.16 (0.14-0.18) per person years of follow up (PYFU). Table 1 summarizes in detail the causes of anti-HIV first-line therapy discontinuation. The most represented adverse events were gastrointestinal symptoms (28.5%), hematological (13.2%) or metabolic (lipid and glucose metabolism, lipodystrophy) (11.3%) toxicities and hypersensitivity reactions (9.3%). By a Kaplan-Meier estimation, the probabilities (95% CI) of being free from an adverse event at 90 days, 180 days, one year, two years, and five years were 0.88 (0.86-0.90), 0.85 (0.83-0.87), 0.79 (0.76-0.81), 0.70 (0.67-0.74), 0.55 (0.50-0.61), respectively. Figure 2 shows the Kaplan-Meier curves overall and stratified for gender, risk group, CDC stage at baseline. The test of proportional-hazard assumption on the data set with the full set of covariates yielded global p-values of 0.3 and 0.8, using the anti-HIV antiretroviral therapy encoding (ii) and (iv), respectively. The proportional hazard hypothesis could not be rejected as well with the other therapy encodings. This allowed the subsequent fit of a main-effect multivariable Cox regression model.

Table 4 reports the relative hazards of the main-effects Cox models using the two different anti-HIV antiretroviral therapy encoding (ii) and (iv). These encodings were the only ones showing significantly different (defined with a p-value<0.05) hazards among such anti-HIV treatment categories, therefore encodings (i) and (iii) did not reveal differences among treatment strata. Factors associated with an increased hazard of discontinuation for adverse events were: an older age, a CDC stage C vs. A, female gender, homo/bisexual risk group as compared to the heterosexual category, HBsAg positivity, zidovudine, stavudine, zalcitabine, didanosine, efavirenz, full dose ritonavir, or indinavir intake, any NRTI+PI/r intake (excluding Kaletra® and Reyataz®) as compared to Truvada® + Sustiva®. The relative hazard (RH) of Atripla® compared to the Truvada® + Sustiva® category was 0.99, 95% CI 0.12-8.44, p=0.99. By changing the reference categories, we found that any NRTI+PI/r combination (excluding Kaletra® and Reyataz®) showed an increased risk of adverse events as compared to any other NRTI+NNRTI intake (not including Truvada®, Sustiva®, or Atripla®), with a RH of 2.49, 95% CI 1.22-5.06, p=0.012. In addition, any NRTI+PI/r combination vs. Kaletra® and Reyataz® yielded a RH of 3.10, 95% CI 1.57-6.09, p= 0.001.

**Table 4:** Multivariable Cox model fit: relative hazards of adverse events, with two different antiretroviral therapy encodings (n=1,096)

**Factor**	**RH**	**95% CI**		**p-value**
		**Lower**	**Upper**	
calendar year (per more recent)	1.01	0.96	1.07	0.6462
gender M vs. F	0.52	0.38	0.71	<0.0001
age per one year older	1.04	1.02	1.05	<0.0001
nationality non-Italian vs. Italian	0.82	0.57	1.17	0.2724
nationality unknown vs. Italian	1.08	0.62	1.87	0.7941
risk homo/bisexual vs. heterosexual	2.03	1.40	2.94	0.0002
risk IDU vs heterosexual	1.16	0.75	1.79	0.5153
risk other/unknown vs. heterosexual	0.74	0.52	1.07	0.1090
years from first positive test	0.99	0.96	1.02	0.3804
CDC stage B vs. A	1.10	0.79	1.54	0.5716
CDC stage C vs. A	1.57	1.14	2.15	0.0051
HBsAg positive vs. negative	1.67	1.04	2.69	0.0356
HBsAg unknown vs. negative	1.11	0.54	2.28	0.7780
HCVAb positive vs. negative	1.02	0.69	1.52	0.9109
HCVAb unknown vs. negative	0.68	0.34	1.36	0.2766
CD4+ per cell/mm^3^ higher	1.00	1.00	1.00	0.8679
HIV-RNA per Log_10_ higher	0.85	0.68	1.07	0.1761
subtype 17 BF vs. B	1.49	0.81	2.75	0.1973
subtype 28 BF vs. B	0.96	0.56	1.65	0.8714
subtype 29 BF vs. B	0.90	0.38	2.15	0.8113
subtype C vs. B	1.87	0.83	4.18	0.1289
subtype F1 vs. B	1.89	0.94	3.83	0.0747
subtype other vs. B	1.46	0.75	2.86	0.2691
subtype unknown vs. B	0.95	0.70	1.29	0.7441
bilirubin high vs. normal	1.02	0.46	2.26	0.9576
bilirubin unknown vs. normal	0.67	0.42	1.06	0.0845
hemoglobin normal vs. low	0.97	0.66	1.40	0.8525
hemoglobin unknown vs. low	0.91	0.47	1.78	0.7903
gammaGT high vs. normal	0.93	0.62	1.39	0.7125
gammaGT unknown vs. normal	1.12	0.77	1.64	0.5507
glucose diabetes vs. normal	1.55	0.80	3.01	0.1959
glucose high vs. normal	1.73	0.92	3.26	0.0888
glucose unknown vs. normal	1.04	0.55	1.96	0.9142
GPT high vs. normal	1.20	0.87	1.66	0.2757
GPT unknown vs. normal	0.70	0.37	1.32	0.2691
HDL normal vs. low	1.04	0.60	1.81	0.8911
HDL unknown vs. low	0.97	0.35	2.67	0.9476
LDL high vs. normal	1.51	0.56	4.03	0.4137
LDL unknown vs. normal	1.62	0.58	4.51	0.3551
TGL high vs. normal	0.95	0.63	1.42	0.8048
TGL unknown vs. normal	1.65	1.11	2.44	0.0125
MDRD normal vs. low	1.41	0.58	3.41	0.4430
MDRD unknown vs. low	1.94	0.69	5.45	0.2098
anti-HCV therapy	0.73	0.22	2.41	0.6107
anti-TB therapy	0.93	0.63	1.37	0.7013
anti-PCP therapy	0.93	0.68	1.27	0.6533
other antibiotic therapy	0.99	0.71	1.37	0.9389
other concomitant drugs	0.76	0.56	1.03	0.0785
Atripla® vs. Truvada®+Sustiva®	0.99	0.12	8.44	0.9901
Reyataz® vs. Truvada®+Sustiva®	0.27	0.03	2.27	0.2270
Kaletra® vs. Truvada®+Sustiva®	1.26	0.53	2.96	0.6025
any other therapy vs. Truvada®+Sustiva®	1.11	0.39	3.20	0.8408
any other NRTI+NNRTI vs. Truvada®+Sustiva®	1.56	0.64	3.83	0.3267
any other NRTI+PI vs. Truvada®+Sustiva®	1.59	0.62	4.04	0.3339
any other NRTI+PI/r vs. Truvada®+Sustiva®	3.89	1.38	10.95	0.0101
Emtricitabine* (in the cART, yes vs. no)	1.19	0.46	3.07	0.7127
Lamivudine* (in the cART, yes vs. no)	1.21	0.70	2.09	0.5004
Abacavir* (in the cART, yes vs. no)	2.33	0.93	5.87	0.0719
Zidovudine* (in the cART, yes vs. no)	3.51	1.34	9.18	0.0107
Stavudine* (in the cART, yes vs. no)	2.84	1.06	7.60	0.0375
Zalcitabine* (in the cART, yes vs. no)	3.47	1.29	9.34	0.0136
Didanosine* (in the cART, yes vs. no)	2.27	1.29	3.98	0.0042
Tenofovir* (in the cART, yes vs. no)	2.00	0.65	6.20	0.2282
Nevirapine* (in the cART, yes vs. no)	1.42	0.67	2.98	0.3586
Efavirenz* (in the cART, yes vs. no)	2.40	1.14	5.03	0.0205
Atazanavir* (in the cART, yes vs. no)	0.15	0.02	1.34	0.0898
Fosamprenavir* (in the cART, yes vs. no)	2.77	0.76	10.09	0.123
Indinavir* (in the cART, yes vs. no)	1.76	1.04	3.00	0.0357
Lopinavir* (in the cART, yes vs. no)	0.55	0.23	1.33	0.1843
Nelfinavir* (in the cART, yes vs. no)	1.37	0.66	2.86	0.396
Ritonavit full dose* (in the cART, yes vs. no)	2.76	1.39	5.52	0.0039
Saquinavir* (in the cART, yes vs. no)	1.75	0.91	3.36	0.0922
Ritonavir boosting dose* (in the cART, yes vs. no)	1.19	0.41	3.44	0.7527

*Substituting anti-HIV therapy encoding (iv) to (ii) and fitting with the other covariates (single drugs’ RH are mutually adjusted). Note that both encoding (i) that compared 2NRTI+1NNRTI, 2NRTI+1PI, 2NRTI+1PI/r, others, and encoding (iii) that compared therapy combinations with a frequency of at least 10, did not show significantly different hazards (at p=0.05 level).

Also, in order to analyze the possible role of the introduction in Italy of tenofovir (November 2002), Truvada® (September 2005), and Atripla® (October 2008), the calendar year was stratified into: 1988–1996, 1997–1998, 1999–2002, 2003–2005, 2006–2008, and 2009–2010. However, this did not lead to any appreciable difference across time periods.

Of note, a trend toward an increased risk of adverse events was observed for HIV genotype F1 (when compared to genotype B). Moreover, unknown TGL showed a higher RH as compared to the corresponding “normal” (<150 mg/dL) category. We also formally tested for interactions between the calendar year and the efavirenz intake, using an ANOVA comparison on the nested models (i.e. with/without interaction), but the more complex model did not show a higher likelihood (p=0.5); the interaction term was not significant and the significant effect of efavirenz was canceled out.

In order to better assess the role of CD4^+^ T cell counts, different Cox regression models were fit with either the sole CD4^+^ or *log*(CD4^+^) cell/mm^3^ count or including the full covariate set with the exception of the CDC stage. Only for the univariable *log*(CD4^+^) regression there was a significant (p<0.05) decreased hazard of an adverse event per *log*(CD4^+^) higher (RH=0.88, 95% CI 0.79-0.99).

As a sensitivity analysis, we repeated the multivariable Cox regression using the subset of therapies posterior to 1998 (n=779, number of events=193, PYFU=1228.948, incidence of 0.16 with a 95% CI of 0.14-0.18), including only cART regimens (although 12/779, i.e. 1.5%, where not 2NRTI+1NNRTI or 2NRTI+1PI±r regimens). The described relative hazards did not change, and in addition there was a significant reduced hazard of an adverse event for any other/unknown risk group as compared to the heterosexual category (RH=0.62, 95% CI 0.40-0.95, p=0.03), and a trend of increased hazard for nevirapine (RH=4.42, 95% CI 0.92-21.12, p=0.06), fosamprenavir (RH=5.64, 95% CI 0.94-34.01, p=0.06), and nelfinavir (RH=4.42, 95% CI 0.97-20.22, p=0.05). Moreover, in this model a significant higher risk of discontinuation for adverse events was demonstrated for HIV genotype F1 (RH 2.63, 95% CI 1.22-5.67, p=0.013 when compared to genotype B), while a trend was observed for genotype C (RH 2.24, 95% CI 0.94-5.33, p=0.067).

## Discussion and conclusion

Since adverse events are the most frequent reason for first-line antiretroviral therapy discontinuation or switch, investigation of variables associated with their occurrence in a routine clinical practice setting is of increasing interest. Such an understanding is crucial to tailor antiretroviral regimens on patients’ characteristics in order to increase the probability of cART tolerability.

In this study several demographic, clinical, laboratory and cART-related variables were investigated, providing useful information for the management of antiretroviral therapy. The prescribed antiretroviral drugs are main determinants of treatment discontinuation for toxicity. As expected, the use of older drugs such as zidovudine, stavudine, zalcitabine, didanosine and full-dose ritonavir was associated with increased risk of adverse events. However, these drugs are no longer recommended for first-line therapy because of their greater potential for toxicity [17]. When considering individual drugs currently recommended as preferred or alternative options in first-line regimens, an higher probability of treatment discontinuation for adverse events was observed for efavirenz (RH 2.4, 95% CI 1.2-5.1, p=0.02). Despite efavirenz has demonstrated high virologic efficacy, both neuropsychiatric and neurocognitive toxicity associated with this drug are not negligible [5,18]. In a recent study, it was shown that nearly 20% of patients discontinue efavirenz due to central nervous system adverse events [19]. Since similar rates of virological response have been observed for other recommended regimens, this observation should be taken into account at the time of prescription of fist-line cART and close clinical monitoring should be warranted in efavirenz-treated patients. However, our analysis includes mainly patients treated with non-co-formulated efavirenz, since the single tablet regimen including efavirenz/tenofovir/emtricitabine is available in Italy from October 2008, and its availability seems to improve treatment convenience and related quality of life [20].

In this study, female patients showed a higher risk of treatment discontinuation for drug-related side effects, in accordance with previous results [3,21] . This might be due to peculiar gender-related pharmacokinetic characteristics which could influence drug exposure [21,22].

An interesting finding is the increased risk of discontinuation for adverse events observed in men who have sex with men. This could be ascribed to a different perception of side effects eventually related to sociocultural barriers, as described among vulnerable populations [23].

Unlike previous studies [2,3], in our population we did not observe a strong relation between discontinuation for toxicity and baseline CD4^+^ cells count or viral load.

Patients harboring HIV genotype F1 demonstrated an higher risk of treatment discontinuation for adverse events and a trend toward an association was observed for genotype C. F1 subtypes have been mostly detected in Latin America [24], where they were reported to be associated with faster HIV progression [25], and Central Africa [26]. F1-carrying patients in our sample were generally immigrants from poor resources countries often with non-steady residence in Italy. This may explain the higher, though non-significant, frequency of interruptions related to F1 subtypes (vs. B).

In agreement with previous studies [3], we confirmed the increased predisposition of older patients to discontinue antiretroviral therapy for side effects. This could be related to several factors, as an altered drug pharmacokinetic (e.g. modification of absorption, protein binding or distribution, impaired drug metabolism) and a potential for drug interactions with co-medications. Since our database does not include detailed data on co-medications other than drugs used for the treatment of opportunistic infections and co-infections, the potential role of drug-drug interactions as a major determinant of treatment discontinuation could not be assessed adequately.

Previous studies suggested that prescription of concomitant medications used for the treatment of opportunistic infections could increase the risk of discontinuation owing to cumulative toxicities or drug interactions [3]. In our population we did not observe an increased risk of discontinuation for adverse events in patients treated for PCP, TB or HCV co-infection. However, patients with AIDS-defining events were more likely to interrupt or switch cART for drug-related side effects.

Co-infection with HBV or HCV have been claimed as a predisposing factor for treatment discontinuation due to hepatic adverse events [9]; however, this association remain controversial [2,3]. In our population we did not observe a significant role of HCV co-infection, but HBsAg-positive patients showed an increased hazard of adverse events. The importance of co-infection with hepatitis viruses could be strictly related to the frequency of prescription of specific antiretroviral drugs, with older drugs bearing a higher risk of hepatotoxicity.

The calendar year was not showing an impact on the hazard of toxicity/allergy events. A similar observation has been previously described in other cohorts [2,27]. This could seem paradoxical since in recent years several drugs with better tolerability profile have been introduced in routine clinical practice. A possible explanation of this finding can rely on the increased number of alternative regimens that have become available in recent years; for this reason clinicians can be more prone to switch drugs also for less severe adverse events. Unfortunately, we could not verify this hypothesis since grading of clinical adverse events was not available in our database.

The potential association of treatment discontinuation for side effects and baseline chemical parameters has not been investigated in previous studies. Chemistry characteristic can represent important tools on the basis of which clinicians can choose the appropriate antiretroviral regimens in order to limit the occurrence of toxicities. In our population, we did not observe any association with the explored parameters; however, the relevant proportion of missing data could have influenced such finding masking the real effects in terms of increased/reduced hazard of adverse events occurrence. Moreover, only baseline markers were analyzed, while the effect of current markers may be crucial, for instance the raise levels of bilirubin during atazanavir-containing antiretroviral therapy [28]. We decided not to perform a time-dependent analysis given the high rate of missing information in the baseline variables. A few multi-centric HIV study cohorts in Italy, specifically the MASTER (http://www.mastercohort.it) and Icona (http://www.fondazioneicona.org) foundations, and in Europe, including the Copenhagen HIV Programme and EuroSIDA (http://www.cphiv.dk) record such detailed information for all patients, besides the standard HIV serologic markers [15,16].

Some limitations should be recognized when interpreting the results of our study. First, cART toxicity was analyzed considering only treatment modification, but clinical events or alterations in laboratory parameters not leading to treatment discontinuation were not investigated, thus leading to an underestimation of overall drug-related side effects. There might be a population bias, since the first-line anti-HIV therapies could have been tuned appositely on the patients’ background, basing on the guidelines available at that time. This can be truer for drug combinations circulating for a longer time, whose adverse effect have been deeply disclosed. Since this study is retrospective, the bias cannot be ruled out even using strong selection criteria and a patient cohort with a high level of detail and follow up.

Another possible limitation of this study is that we conducted a cause-specific analysis, ignoring the potential effects of competing events in the survival analysis; such events may drive the happening of adverse effects, and surely a design of a competing-risk analysis after this study is warranted.

Given the current, fair, rate of virologic and immunologic successes of first-line anti-HIV antiretroviral therapies in the European Union and other developed countries [29-32], along with the decrease of drug-resistance prevalence [33-39], the objective is now the prolongation of the therapy duration with the minimization of adverse events. Intolerance/toxicity still remains the major cause of drug discontinuation in Italy [40]. Therefore, an effort in gathering data from all available sources to assess more precisely prognostic factors and relative hazards of adverse events is warranted. Eventually, a personalized scoring system might be inferred, as it has been done with the prediction of virologic responses and time to virologic failure [41-44].

In conclusion, several predictors of treatment discontinuation for drug-related adverse events were investigated. These findings could help clinicians to identify individuals at higher risk of developing toxicity, thus allowing an improved prescription of antiretroviral regimens tailored on patients’ characteristics. Moreover, these results suggest the importance of anticipating the probability of occurrence of adverse events in order to ensure close clinical and laboratory monitoring and adequate management of side effects which could improve the durability of cART.

## Abbreviations

cART: Combination antiretroviral therapy; CI: confidence intervals; CUSH: Catholic University of the Sacred Heart; gammaGT: gamma-glutamyltransferase; GPT: Glutamate pyruvate transaminases; GFR: Glomerular filtration rate; HDL: High-density lipoprotein; LDL: Low-density lipoprotein; MDRD: Modification of diet in renal disease formula; NNRTI: Non-nucleoside reverse transcriptase inhibitor; NRTI: Nucleoside reverse transcriptase inhibitors; PCP: *Pneumocystis jirovecii* pneumonia; PI: Protease inhibitor; PI/r: PI boosted with ritonavir; PYFU: Person years of follow up; RH: Relative hazard; TB: Tuberculosis; TGL: Triglycerides; 95% CI: 95% Confidence Interval.

## Competing interests

MF received speakers’ honoraria from Abbott Virology, Merck Sharp and Dohme and Janssen-Cilag. MZ received speakers’ honoraria from Abbott Virology, Merck Sharp and Dohme, Janssen-Cilag, Bristol-Myers Squibb and Gilead Science. MC was an employee of Bristol-Myers Squibb from May 2010 to February 2011 and resigned before starting the present work. RC was advisor for Gilead and Janssen-Cilag, received speakers’ honoraria from ViiV, Bristol-Myers Squibb, Merck Sharp and Dohme and Janssen-Cilag, and research support from “Fondazione Roma”. SDG received speakers’ honoraria from ViiV, Bristol-Myers Squibb, Merck Sharp and Dohme and Janssen-Cilag. All other authors: none to declare.

## Authors’ contributions

MCFP contributed to study design, data analysis and interpretation, and article writing; MF and MZ contributed to interpretation of data and article writing; IF, MC, AM, ADA, AB contributed to data collection; RC and SDG coordinated the project and contributed to interpretation of data. All authors reviewed the manuscript during preparation, provided critical feedback and approved the final manuscript.

## Pre-publication history

The pre-publication history for this paper can be accessed here:

http://www.biomedcentral.com/1471-2334/12/296/prepub

## Supplementary Material

Additional file 1 Distribution of first-line anti-HIV therapies by calendar year. Click here for file
