# Supplementary material for: Predictors of first-line antiretroviral therapy discontinuation due to drug-related adverse events in HIV-infected patients: a retrospective cohort study
Source: BMC Infect Dis. 2012 Nov 12;12:296. doi: 10.1186/1471-2334-12-296 (PMC3519703; doi:10.1186/1471-2334-12-296)
Supplement: Additional file 1 — Distribution of first-line anti-HIV therapies by calendar year. [file 1471-2334-12-296-S1.docx]

**Supplementary Data**

**Supplementary Table 1.** Distribution of first-line anti-HIV therapies by calendar year.

| year | Encoding (iv) | | | | | | | | Encoding (i) | | | |
| --- | --- | --- | --- | --- | --- | --- | --- | --- | --- | --- | --- | --- |
|  | Truvada + Sustiva | Atripla | Reyataz ± ritonavir + backbone | Kaletra + backbone | Any other NRTI +NNRTI cART | Any other NRTI +PI cART | Any other NRTI +PI/r cART | Other or subopt | 2NRTI +1NNRTI cART | 2NRTI +1PI cART | 2NRTI +1PI/r cART | Other or subopt |
| 1988 | 0 | 0 | 0 | 0 | 0 | 0 | 0 | 1 | 0 | 0 | 0 | 1 |
| 1989 | 0 | 0 | 0 | 0 | 0 | 0 | 0 | 9 | 0 | 0 | 0 | 9 |
| 1990 | 0 | 0 | 0 | 0 | 0 | 0 | 0 | 18 | 0 | 0 | 0 | 18 |
| 1991 | 0 | 0 | 0 | 0 | 0 | 0 | 0 | 21 | 0 | 0 | 0 | 21 |
| 1992 | 0 | 0 | 0 | 0 | 0 | 0 | 0 | 19 | 0 | 0 | 0 | 19 |
| 1993 | 0 | 0 | 0 | 0 | 1 | 0 | 0 | 18 | 1 | 0 | 0 | 18 |
| 1994 | 0 | 0 | 0 | 0 | 0 | 0 | 0 | 11 | 0 | 0 | 0 | 11 |
| 1995 | 0 | 0 | 0 | 0 | 0 | 0 | 0 | 21 | 0 | 0 | 0 | 21 |
| 1996 | 0 | 0 | 0 | 0 | 1 | 9 | 0 | 25 | 1 | 8 | 0 | 26 |
| 1997 | 0 | 0 | 0 | 0 | 0 | 45 | 0 | 34 | 0 | 43 | 2 | 34 |
| 1998 | 0 | 0 | 0 | 0 | 6 | 60 | 0 | 18 | 7 | 55 | 2 | 20 |
| 1999 | 0 | 0 | 0 | 0 | 22 | 36 | 0 | 1 | 22 | 35 | 0 | 2 |
| 2000 | 0 | 0 | 0 | 1 | 10 | 28 | 2 | 4 | 10 | 28 | 3 | 4 |
| 2001 | 0 | 0 | 0 | 5 | 16 | 19 | 4 | 4 | 16 | 20 | 8 | 4 |
| 2002 | 0 | 0 | 0 | 24 | 15 | 5 | 1 | 5 | 15 | 5 | 25 | 5 |
| 2003 | 1 | 0 | 0 | 35 | 30 | 8 | 1 | 1 | 31 | 8 | 36 | 1 |
| 2004 | 0 | 0 | 0 | 42 | 21 | 7 | 0 | 1 | 21 | 7 | 41 | 2 |
| 2005 | 3 | 0 | 1 | 62 | 20 | 3 | 1 | 3 | 23 | 3 | 63 | 4 |
| 2006 | 12 | 0 | 4 | 50 | 15 | 1 | 1 | 0 | 27 | 1 | 55 | 0 |
| 2007 | 9 | 0 | 3 | 68 | 3 | 0 | 0 | 2 | 12 | 0 | 70 | 3 |
| 2008 | 11 | 0 | 5 | 63 | 1 | 0 | 5 | 0 | 12 | 1 | 72 | 0 |
| 2009 | 9 | 10 | 8 | 38 | 3 | 0 | 0 | 1 | 22 | 0 | 45 | 2 |
| 2010 | 2 | 4 | 1 | 1 | 1 | 0 | 3 | 3 | 7 | 0 | 4 | 4 |
